# Brief intervention to prevent hazardous drinking in young people aged 14–15 in a high school setting (SIPS JR-HIGH): study protocol for a randomized controlled trial

**DOI:** 10.1186/1745-6215-13-166

**Published:** 2012-09-13

**Authors:** Stephanie O’Neil, Simon Coulton, Paolo Deluca, Mark Deverill, Colin Drummond, Eilish Gilvarry, Erin Graybill, Christine Harle, Denise Howel, Eileen Kaner, Paul McArdle, Elaine McColl, Ruth McGovern, Chris Speed, Elaine Stamp, Les Tate, Dorothy Newbury-Birch

**Affiliations:** 1 Institute of Health & Society, Baddiley-Clark Building, Newcastle University, Richardson Road, Newcastle upon Tyne NE2 4AX, UK; 2 Centre for Health Services Research, University of Kent, The Registry, Canterbury, Kent, CT2 7NZ, UK; 3 Addictions Department, Institute of Psychiatry, King’s College London, PO48, 4 Windsor Walk, Denmark Hill, London, SE5 8BB, UK; 4 Northumberland, Tyne and Wear NHS Foundation Trust, St. Nicolas Hospital, Gosforth, Newcastle upon Tyne, NE3 3XT, UK; 5 Newcastle Clinical Trials Unit, Institute of Health & Society, Baddiley-Clark Building, Newcastle University, Richardson Road, Newcastle upon Tyne, NE2 4AX, UK; 6 Young People’s Drug and Alcohol Department, North Tyneside Council, Hudson Street, North Shields, Tyne and Wear, NE30 1DL, UK

**Keywords:** Alcohol, Screening and brief intervention, Feasibility pilot trial, Motivational interviewing, Young people

## Abstract

**Background:**

Whilst the overall proportion of young people drinking alcohol in the United Kingdom has decreased in recent years, those who do drink appear to drink a larger amount, and more frequently. Early and heavy drinking by younger adolescents is a significant public health problem linked to intellectual impairment, increased risk of injuries, mental health issues, unprotected or regretted sexual experience, violence, and sometimes accidental death, which leads to high social and economic costs. This feasibility pilot trial aims to explore the feasibility of delivering brief alcohol intervention in a school setting with adolescents aged 14 and 15 and to examine the acceptability of study measures to school staff, young people and parents.

**Methods and design:**

Seven schools across one geographical area in the North East of England will be recruited. Schools will be randomly allocated to one of three conditions: provision of an advice leaflet (control condition, *n* = 2 schools); a 30-minute brief interactive session, which combines structured advice and motivational interviewing techniques delivered by the school learning mentor (level 1 condition, *n* = 2 schools); and a 60-minute session involving family members delivered by the school learning mentor (level 2 condition, *n* = 3 schools). Participants will be year 10 school pupils (aged 14 and 15) who screen positively on a single alcohol screening question and who consent to take part in the trial. Year 10 pupils in all seven schools will be followed up at 6 and 12 months. Secondary outcome measures include the ten-question Alcohol-Use Disorders Identification Test. The EQ-5D-Y and a modified short service use questionnaire will inform the health and social resource costs for any future economic evaluation.

Young people recruited into the trial will also complete a 28-day timeline follow back questionnaire at 12-month follow-up. A qualitative evaluation (with young people, school staff, learning mentors, and parents) will examine facilitators and barriers to the use of screening and brief intervention approaches in the school setting in this age group.

**Trial registration:**

Trial reference number ISRCTN07073105

## Background

Although the proportion of young people in England aged between 11 and 15 years who report that they have drunk alcohol decreased from 62% to 51% between 1988 and 2009, the mean amount consumed rose from 6.4 to 11.6 units of alcohol per week over a similar period [1]. Approximately 33% of 15 to 16 year olds in England reported alcohol intoxication in the previous month [2] with adolescents in the UK being amongst the heaviest drinkers in Europe [3], leading to high social and economic costs [4].

A recent review of preventive interventions to reduce the harm associated with adolescent substance use outlined the positive potential of brief alcohol intervention [5]. Brief intervention is secondary preventive activity, aimed at individuals whose alcohol consumption level or pattern is likely to be harmful to their health or well-being [6]. Brief interventions generally consist of structured advice or counselling of short duration, which is aimed at reducing alcohol consumption or decreasing the number or severity of problems associated with drinking [7].

Although there is a large volume of evidence on primary prevention, which aims to delay the age that drinking begins and which uses general health education to prevent underage drinking, this body of work has been reported to be methodologically weak [8] and only a relatively small number of programmes have reported positive outcomes [9]. Thus, targeting interventions at young people who are already drinking alcohol is likely to be a more effective strategy, since the intervention will have more relevance for the individuals receiving them.

A key feature of brief intervention is that it is designed to be delivered by generalist practitioners (not addiction specialists) and targeted at individuals who are generally not experiencing severe problems, such as alcohol dependence, and who may not be aware that they are experiencing alcohol-related problems. Thus, the goal is usually reduced alcohol consumption or a decrease in alcohol-related problems [10].

Although there is variation in the duration and frequency of brief alcohol intervention [11], many approaches are based on motivational interviewing. This is a client-centred, directive approach, which seeks to elicit behaviour change by helping individuals to explore and resolve ambivalence about reducing alcohol consumption. This approach aims to resolve conflicts regarding the pros and cons of behaviour change and thus enhance motivation. Motivational interviewing is characterized by empathy and an avoidance of direct confrontation. Elicited statements associated with positive behaviour change are encouraged, so as to support self-efficacy and a commitment to take action [12]. With young people, however, motivational interviewing is still being developed and adaptations need to be considered for different age groups.

Meta-analyses have consistently reported that college and university students who received brief interventions subsequently reduced their drinking behaviour compared with controls who typically received assessment only [13,14]. The key elements of the brief interventions were personalized feedback on alcohol consumption, typically with a normative component [14] or motivational interviewing approaches. Such brief interventions usually achieved small to medium effects [15] across multiple measures of alcohol consumption including quantity, frequency, and quantity of drinking. The effects of brief interventions on drinking behaviour often peaked in the shorter term (generally 6 months) then diminished over time [13]. However, reductions in alcohol-related problems often took longer to emerge but were found in longer-term follow-up (12 to 18 months). Hence, it is important to have brief intervention outcomes measuring both consumption and alcohol-related problems and to follow up participants after both shorter and longer times.

There is, however, insufficient evidence to support confidence about the use of brief intervention to reduce excessive drinking or alcohol-related harm in younger adolescents. Nevertheless, the current evidence base suggests that the most effective forms of brief intervention are those containing personalized feedback about a young person’s drinking behaviour and motivational interviewing approaches to help reduce levels of alcohol-related risk.

This work builds on the evidence base by focusing on screening and brief intervention to reduce hazardous drinking in younger adolescents (aged 14 and 15). Hazardous drinking among young people commonly occurs in the context of other forms of ‘disinhibitory behaviour’, such as aggression and risk-taking [16]. Whilst these behaviours are well known to be linked, it is not clear whether drinking leads to these behavioural problems or whether there are common causal factors [17]. Nevertheless, it is possible that if a brief intervention is effective at reducing hazardous drinking, it might also result in a range of other positive behavioural outcomes. A significant positive association between alcohol dose and aggression for both sexes has been found [18], and a study of US accident and emergency attendees showed reductions in both aggression and alcohol misuse following a brief alcohol intervention [19]. For this reason, in addition to measuring alcohol use, we have included a range of behavioural measures as study outcomes.

Moreover, there is some evidence in the UK that parents’ drinking behaviour and attitudes about alcohol may shape their children’s views about drinking, particularly in younger children, [20] and it would be advantageous to include parents in brief advice sessions with young people about alcohol [21]. However, it may be difficult to get both children and parents to agree to such sessions. This feasibility pilot trial will include two brief intervention conditions: one that only involves young people and one that includes young people and their parents.

The Medical Research Council (MRC) has presented a framework for the evaluation of complex interventions [22]. This work represents the development and piloting phases of that framework. Conducting a full-scale randomized control trial (RCT) and economic evaluation of screening and brief intervention versus ‘standard care’ in this population is likely to need the involvement of many schools and to be resource intensive. As there are uncertainties regarding rates of eligibility, consent, participation in the intervention, and retention for follow-up, and regarding the feasibility and acceptability of the intervention for a range of stake-holders (teachers, learning mentors, young people, and parents) we deem that this feasibility pilot trial is essential to inform the design and conduct of a larger-scale study [23,24].

### Aim of the study

To assess the feasibility of a RCT of screening and brief alcohol intervention (in a school setting) to reduce hazardous drinking in adolescents.

### Objectives

• To conduct a three-arm feasibility pilot trial (with randomization at school level) to assess the feasibility of a future large-scale randomized controlled trial of brief alcohol intervention in a school setting.

• To explore the feasibility and acceptability of brief alcohol intervention and study measures to staff, young people, and parents.

• To explore the fidelity of the interventions as delivered by school-based learning mentors.

• To estimate the parameters for the design of a definitive trial of brief alcohol intervention, including rates of eligibility, consent, participation, and retention at 6 and 12 months.

• To pilot the collection of cost and resource use data to inform cost effectiveness and utility analysis in a definitive trial.

• To develop the protocol for a definitive trial and economic evaluation of the impact of brief alcohol intervention compared with standard advice to reduce alcohol consumption.

## Methods/design

### Setting

Seven schools across one geographical area in the North East of England will be recruited. Approximately 1,500 young people and their parents will be contacted, via letter, by the study organizers and invited to take part. All high schools, catering for pupils aged 11 to 16, will be eligible to take part. The study catchment area enables broad population coverage; randomization procedures will ensure that each study condition is adequately weighted by numbers of participants and socio-economic status using appropriate markers (school size and proportion of students receiving free school meals).

### Staff delivering interventions

Local areas vary in their entry requirements for learning mentors. However, as a minimum, they need to have a good standard of general education, especially literacy and numeracy, as well as experience of working with young people. Learning mentors are specifically trained to provide a complementary service to teachers and other staff, addressing the needs of children who require assistance in overcoming barriers to learning in order to achieve their full potential. Learning mentors work with a range of pupils, but give priority to those who need the most help, especially those experiencing multiple disadvantages. Mentoring covers a wide range of issues, from punctuality, absence, bullying, challenging behaviour and abuse to working with able and gifted pupils who are experiencing difficulties. Learning mentors are therefore well-placed within a school setting to deliver the intervention.

### School site recruitment

Contact with each school site will initially be made by telephoning and emailing the school office and securing appropriate points of contact, such as the head teacher or deputy head teacher (either of year 10 or of the whole school) and members of staff responsible for Personal Social and Health Education or pastoral care. Visits will then be arranged to allow research staff to explain the feasibility pilot trial protocol, secure staff consent to participate in the trial and to organize screening of all year 10 pupils and learning mentor training. Final approval will be secured from head teachers at each school.

### Participants

Participants will be young people aged 14 and 15 in year 10 at schools in the North East of England.

### Inclusion criteria

Young people aged 14 or 15, scoring positive on the Adolescent Single Alcohol Question (A-SAQ) which is a modified version of the M-SASQ [25], with quantity and frequency measures adjusted to reflect guidelines for an adolescent population [26]. Young people also need to be willing and able to provide informed consent for intervention and follow-up, which will be assessed by the learning mentor.

### Exclusion criteria

Young people already seeking help for an alcohol-use disorder, receiving support from child and adolescent mental health services, or whose parents do not wish for them to take part will not be eligible to take part in the study.

### Randomization

Each of the seven schools will be allocated at random to one of three intervention conditions: provision of an advice leaflet that gives contact details for local services (control condition, *n* = 2 schools); a 30-minute session of structured advice (level 1 condition, *n* = 2 schools); and a 60-minute session involving family members (level 2 condition, *n* = 3 schools). Allocation of schools to intervention will be conducted by the study statistician, taking school size and socio-economic factors (proportion of free school meals) into account.

### Consent

**Figure 1:**
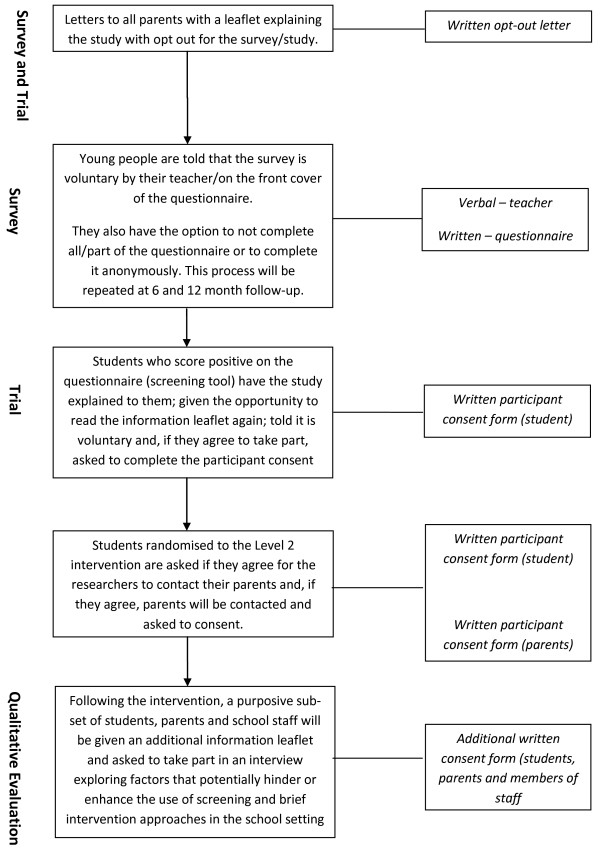
Flow chart of consent (survey and trial).

Consent to participate will be obtained in a three-stage process (Figure 1). First, in advance of screening, all parents and caregivers will be informed by letter that screening for alcohol use and the later study will be taking place in school. Parents’ names and addresses will be provided by the schools. This letter will be posted directly via Royal Mail to parents by the research team or the school directly and will include a prepaid return envelope, addressed to the research team at the university, and a study information leaflet. Parents will have the option to indicate that they do not wish for their children to be screened or considered for participation in the study at this stage by completing and returning an opt-out form.

Prior to completing the screening questionnaire, young people will also be given the opportunity to opt out of the study by putting the questionnaire back into the envelope uncompleted. This will be made clear both verbally (by the member of school staff overseeing completion of questionnaires) and in written form (clear instructions on the front cover of the questionnaire). Obtaining young people’s consent to take part in this way, passive rather than written consent, is a method widely used in various national youth surveys of alcohol consumption and other health behaviours; such as those conducted by the NHS Information Centre, which annually surveys drinking and drug use by young people aged 11 to 15 in England and Wales [2].

Finally, those who then screen positive on the screening questionnaire will have the feasibility pilot trial explained to them verbally by learning mentors, who will obtain written consent from young people only (control and level 1) and from young people and parents (condition 2 only).

### Screening

Young people will complete a health and lifestyle questionnaire administered during a predefined school lesson. In all conditions, the research team will provide support to school staff in implementing screening systems tailored to the needs of a school setting.

The envelope will contain a series of questionnaires including the A-SAQ, ‘In the last 6 months how often have you drunk more than three units of alcohol?’ with the response options of ‘Never; less than four times; four or more times but not every month; at least once a month but not every week; every week but not every day; every day’. Scoring ‘four or more times’ or more frequently indicates a positive screen and therefore indicates that the young person is eligible to take part in the trial. The screening questionnaire includes illustrations to define a unit of alcohol [27].

The pack of questionnaires to be completed will also include a general lifestyle questionnaire addressing a number of areas (diet, smoking, exercise, and alcohol consumption) and the 14-item Warwick-Edinburgh Mental Well-Being Scale (WEMWBS) to assess general psychological health [28]. Alcohol-use frequency, quantity (on a typical occasion) and binge drinking will also be assessed using the modified ten-question Alcohol-Use Disorders Identification Test (AUDIT) [29] and the scores compared with the answers on the A-SAQ. Alcohol-related problems will be assessed using the validated Rutgers Alcohol Problems Inventory (RAPI), which includes measures on aggression [30]. The EQ-5D-Y, which is a recently developed child-friendly version of the EQ-5D, will be used to assess health utility scores [31], and a modified short service use questionnaire (S-SUQ) will inform the health and social resource costs for any future economic evaluation [32]. Demographic information will be collected, including sex and ethnicity, as well as contact information, participants’ names, and the names of the school, class, and teacher responsible for Personal, Social and Health Education (PSHE).

To ensure anonymity, students will then be asked to put their questionnaire into an unmarked envelope, which they themselves will seal and place in an open box at the front of the class. It will be made clear to the young person after completing the questionnaire that only the research team will have access to this information. However, for those who have completed their personal details, the students will be told that their names may be given to a learning mentor at their school. It will be made clear that learning mentors will not see the completed questionnaire but will know that the young person has scored positively on the A-SAQ. Finally, the class teacher will give all young people a healthy living leaflet and a £5.00 retail gift voucher.

### Study intervention

The three-armed cluster randomized controlled feasibility pilot trial incorporates a control condition and two intervention conditions. The recipients of these interventions, the feasibility pilot trial participants, will be the young people who screen positively for alcohol misuse using the alcohol screening questionnaire and who consent to take part in the study.

#### Control condition (Arm A)

Participants from the schools allocated to the control condition will be provided with an alcohol advice leaflet during an individual appointment with a learning mentor at their school. They will also continue to receive ‘standard alcohol advice’, as delivered as part of the school curriculum in PSHE lessons.

#### Level one intervention (Arm B)

In addition to PSHE, participants from the schools allocated to the level one intervention will take part in a 30-minute personalized session delivered by a learning mentor (at school), which includes structured feedback about their drinking behaviour and advice about the health and social consequences of continued hazardous alcohol consumption. The brief intervention utilizes the technique of motivational interviewing [33] and encompasses the elements of the FRAMES approach for eliciting behaviour change (feedback, responsibility, advice, menu, empathy, and self-efficacy) [34]. The young people will also receive the same alcohol advice leaflet as those in the control group.

#### Level two intervention (Arm C)

In addition to PSHE and the level one intervention, participants from schools allocated to the level two intervention will be invited to attend a subsequent 60-minute session (facilitated by a learning mentor), which will occur either during or after school hours, either within the school or in a community centre nearby, and which will have parental or family involvement (either one or both parents or another carer or family member). This session will only take place if the young person consents to parental involvement and parents subsequently agree to take part; however, using an intention-to-treat approach, the case will be entered into the trial based on the young person agreeing to the level one intervention. Again, this intervention utilizes the technique of motivational interviewing [33]. It aims to explore the young person’s motivation to change drinking behaviour and the family’s motivation to facilitate and support change. It is anticipated that this session will result in a ‘mutual agreement’ or ‘family action plan’ between the young person and family members present regarding the young person’s alcohol consumption.

In all conditions, any learning mentor who has any concerns about the welfare of the young person involved will follow the school’s policy and procedures for reporting of safeguarding concerns [35].

### Follow-up

The entire cohort of year 10 pupils at the seven schools will be followed up at 6 and 12 months. Young people will be screened using the same method and series of questionnaires as at baseline. In addition to the screening questionnaire completed by all year 10 pupils, all those who have consented to the feasibility pilot trial, in all conditions, will have a one-to-one appointment arranged with a learning mentor to complete a timeline follow back (TLFB) questionnaire at the 12 month follow-up point, when the young people will have started the next school year (year 11).

### Training and support

All learning mentors will only receive school-based training in the study procedures and the intervention relevant to their school (control, level 1, or level 2). Training for learning mentors will be manual guided and divided into two half-day sessions. The first session will introduce the feasibility pilot trial; examine alcohol-based issues and explore what is involved in taking part in a research project. The second session outlines the steps involved in delivering the intervention to young people.

Learning mentors will be brought together at one of the schools (or an appropriate local site) for this training. Such outreach training was found to be the most cost-effective implementation strategy for alcohol screening and brief intervention delivery in other settings [36]. Training for learning mentors will be carried out by an experienced trainer using a simulated subject scenario within a training package developed and employed in other studies of brief interventions [25,37,38]. Learning mentors will be provided with support materials and on-going support and supervision on implementing screening; paperwork relevant to the research will be provided by the research team, who will also act as the site study coordinator. Learning mentors will record all time spent on the project using a case diary, which will be used as part of the economic analysis. Research staff and trainers will maintain regular contact with schools throughout the study period, including site visits and telephone support.

#### Fidelity to intervention

An important measure of the process relates to how the intervention is conducted. A convenience sample of one interaction carried out per learning mentor (with learning mentors, young people, and parents who consent) will be digitally voice recorded and assessed for treatment fidelity by two independent expert raters from the research team using the BECCI rating scale [39]. Potential participants will be informed that participation is not compulsory. All information relating to the names of the participants or any identifiable features will not be transcribed and the interview will be identified by case number.

### Financial incentives

Each school site will receive a £1,000 payment to cover the burden of having the research take place.

### Participant incentives

All young people who hand in an envelope with the questionnaire, whether completed or not, at baseline will be given a £5.00 retail gift voucher. Provision of a one-off gift voucher is designed to be appreciative and to act as compensation for the time and inconvenience of research participation.

### Qualitative evaluation of the feasibility/pilot trial

Following the intervention, young people, parents, and school staff will be given an additional information leaflet and asked if they would consent to take part in a semi-structured interview. Potential participants will be informed that participation is not compulsory. Interviews will be conducted with a purposive sample of 47 made up of (i) teachers and mentors and (ii) young people and parents, to explore facilitators and barriers to the use of screening and brief intervention approaches in the school setting with the target age group.

The interviews with teachers and learning mentors will explore the feasibility of implementation of screening and interventions in a school setting, including: prioritization of educational or well-being work; the scope for team or individual professional input; staff skill mix and turnover; resources; role development and training needs; and participants’ consent. The interviews with participants and their parents will explore acceptability of screening and brief alcohol intervention in the school setting, including: consent procedures; parental involvement in consent or intervention; the comprehensibility and burden of study measures and follow-up procedures; and the appropriateness of school-led health promotion work across the school-home interface.

### Economic evaluation

The health economic analyses will describe the costs of introducing and running the brief intervention and will focus on examining what resource data we should collect from the schools (that is, learning mentor time, including training); and what NHS and social care data we should collect (and how) in terms of on-going staff and capital costs, as well as capturing any potential cost saving or increase in NHS and social care resource use in a definitive trial to assess the possible cost effectiveness of the intervention.

### Planned analysis

#### Statistical analysis

**Figure 2:**
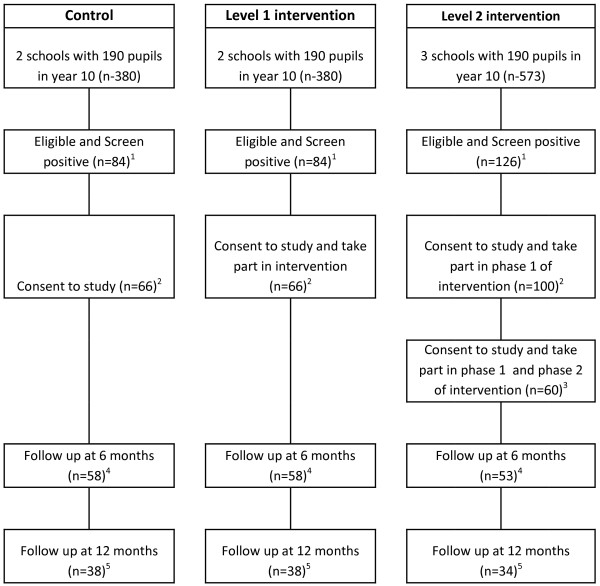
Flow chart for SIPS JR-HIGH.

This is a feasibility pilot trial and, therefore, a formal power calculation is not required. However, providing data for the power calculation of a definitive trial is an important function of a pilot study; a minimum number of 30 participants per group at follow-up is recommended to estimate a parameter for this purpose [23]. Our estimates suggest that this should be more than achieved if all pupils in year 10 in seven schools are invited to take part (Figure 2).

The statistical analyses will be primarily descriptive, providing an estimate of eligibility, recruitment, intervention delivery, and retention rates in the study population. These key feasibility pilot trial parameters will inform the power calculation for a future definitive trial and confirm other aspects of trial design (in particular, the acceptability of study processes and outcome measure to young people, their parents, teachers, and learning mentors). Data pertaining to the flow of participants through the study will be ascertained and include numbers screened, prevalence of the target condition (that is, numbers screening positive on the A-SAQ), numbers providing contact details, numbers eligible and willing to consent, and numbers followed up successfully at 6 and 12 months. In addition we will ascertain data completeness of the instruments and any potential bias in the completion of follow-up data to inform the choice of instruments in a future definitive trial.

Variability in the primary outcome of the proposed definitive trial (total consumption at 12 months using the TLFB-28 within intervention groups) will be combined with recruitment and response rates and estimates of the intraclass correlation coefficient to plan the necessary sample size for a definitive study.

#### Qualitative analysis

We will aim for a maximum variation sample to achieve a broad perspective on the issues being explored. Sampling criteria will be: school or area; intervention condition; participant type (teacher, mentor, pupil, and parent); and sex. Emergent issues from earlier interviews will be explored in subsequent interviews and the total number of interviews will be determined by data saturation (no new issues or themes emerging from within or across participants). All interviews will be audio-tape recorded and transcribed verbatim. Analysis will be conducted using a structured thematic approach to code, classify, and organize interview content systematically into key themes. Analysis will be conducted using QSR Nu*Dist software to assist systematic coding in identifying emerging patterns between staff roles and centres.

### Ethical and research governance approval

The research study has been granted ethical approval by Newcastle University (Reference 0508), who will act as a sponsor for the research. Approval has also been granted by North Tyneside Council. This feasibility pilot trial is funded by the National Institute for Health Research (NIHR). Trial reference number ISRCTN07073105.

The feasibility pilot trial will be managed through a central co-ordinating team. The programme management group (PMG) will be responsible for ensuring the appropriate, effective and timely implementation of the SIPS JR-HIGH Pilot Trial.

A trial steering group (TSG) will be appointed and will concentrate on progress of the feasibility pilot trial against projected rates of recruitment and retention, adherence to the protocol, participant safety, and the consideration of new information of relevance to the research question. Written charters will be agreed and used by the PMG and TSG.

## Discussion

It is important to perform pilot RCTs when the logistics of a large-scale trial are unclear [23,24]. Whilst the use of brief intervention for adults is established in a health setting [11], and there is evidence of their effectiveness in college and university students [13,14], there has been very little work in the UK exploring the early identification (screening) and brief intervention to reduce risky drinking in younger adolescents (aged 11 to 15), making a pilot study a necessary and important step of a definitive evaluation. Learning mentors have a distinct role in supporting and nurturing young people within schools; however, their time is considerably constrained. This work will explore whether young people, and indeed learning mentors, feel that learning mentors are the right people in this setting to carry out brief interventions over alcohol use. The findings of this study will also contribute to the wider understanding of carrying out brief interventions with young people by indicating how such a brief intervention is likely to be received in the school setting by young people, learning mentors, school staff, and parents.

Finally, the findings from this study will indicate whether and how a definitive trial can establish the effectiveness and cost effectiveness of alcohol screening and brief intervention in a school setting. The outcomes will include a protocol for such a trial, with a sample size calculation, which will usefully extend the evidence base in this field at an international level.

## Trial status

### Project timescales

The feasibility pilot trial duration is 22 months and started in October 2011. Recruitment will take place until July 2012.

## Abbreviations

A-SAQ: Adolescent–Single Alcohol Question; AUDIT: Alcohol-Use Disorders Identification Test; EQ-5D-Y: European Quality of Life-5 Dimensions-Youth; FRAMES: Feedback, responsibility, advice, menu, empathy and self-efficacy; M-SASQ: Modified–Single Alcohol Screening Questionnaire; NIHR: National Institute for Health Research; PHR: Public Health Research; PSHE: Personal, social and health education; PMG: Programme management group; RAPI: Rutgers Alcohol Problems Inventory; RCT: Randomized controlled trial; S-SUQ: Short service use questionnaire; TLFB: Timeline follow back; TSC: Trial steering group; WEMWBS: Warwick-Edinburgh Mental Well-Being Scale.

## Competing interests

The authors declare that they have no competing interests.

## Authors’ contributions

All of the authors contributed to the design and development of this feasibility pilot trial protocol. DNB is the chief investigator and SO’N is the project manager of the SIPS JR-HIGH feasibility pilot trial. The disciplines represented in the team include: public health research (EK, DNB, SO’N, RM); alcohol and policy expertise (EK, DNB, SO’N, RM); conducting research with children and young people (SO’N, RM); child and adolescent psychiatry (PM); health psychology (PD); addiction psychiatry (CD, EG); criminology (DNB); medical statistics (DH, SC, ES) and trial methodology (EM, SC, EK, CD, DH, CS, CH), health economics (MD, EG), and trial management (CS, CH). LT has experience of education and learning mentors. The Newcastle Clinical Trials Unit is a UK CRC-registered Clinical Trials Unit, with a strong track record in the design, conduct, and analysis of NIHR-funded trials, including those of complex interventions and feasibility/pilot trials. SO’N wrote the first draft of the paper and all authors contributed to successive drafts. All authors read and approved the final manuscript.

## References

[B1] NHS Information Centre Smoking, Drinking and Drug Use Among Young People in England in 2009 2010 London: Health and Social Care Information Centre

[B2] Fuller E Drinking and Drug Use Among Young People in England in 2008 2008 London: NHS Information Centre for Health and Social Care

[B3] Hibbell B Guttormsson U Ahlstrom S Balakireva O Bjarnason T Kokkevi A Kraus L The 2007 ESPAD Report: Substance Use Among Students in 35 European Countries 2009 Sweden: ESPAD

[B4] Rehm J Mathers C Popova S Thavorncharoensap M Teerawattananon Y Patra J Global burden of disease and injury and economic cost attributable to alcohol use and alcohol-use disorders Lancet 2009 373 2233 2233 10.1016/S0140-6736(09)60746-7 19560604

[B5] Toumbourou J Stockwell T Neighbors C Marlatt G Sturge J Rehm J Interventions to reduce harm associated with adolescent substance use Lancet 2007 369 1391 1401 10.1016/S0140-6736(07)60369-9 17448826

[B6] Kaner E Newbury-Birch D Heather N Miller P Brief interventions Evidence-based Addiction Treatment. Part 3 Treatment Methods 2009 10 San Diego, California: Elsevier

[B7] Kaner E Dickinson H Beyer F Pienaar E Schlesinger C Campbell F Saunders J Burnand B Heather N The effectiveness of brief alcohol interventions in primary care settings: a comprehensive review Drug Alcohol Rev 2009 28 301 323 10.1111/j.1465-3362.2009.00071.x 19489992

[B8] Foxcroft DR Lister-Sharp D Lowe G Alcohol misuse prevention for young people: a systematic review reveals methodological concerns and lack of reliable evidence of effectiveness Addiction 1997 92 5 531 537 10.1111/j.1360-0443.1997.tb02911.x 9219376

[B9] Foxcroft DR Ireland D Lister-Sharp DJ Lowe G Breen R Longer-term primary prevention for alcohol misuse in young people: a systematic review Addiction 2003 98 397 411 10.1046/j.1360-0443.2003.00355.x 12653810

[B10] Bien TH Miller WR Tonigan JS Brief interventions for alcohol problems: a review Addiction 1993 88 3 315 335 10.1111/j.1360-0443.1993.tb00820.x 8461850

[B11] Kaner E Beyer F Dickinson H Pienaar E Campbell F Schlesinger C Heather N Saunders J Burnand B Effectiveness of brief alcohol interventions in primary care populations Cochrane Database Syst Rev 2007 2 CD004148 004110.001002/14651858.CD14654148.pub14651853 17443541 10.1002/14651858.CD004148.pub3

[B12] Emmons KM Rollnick S Motivational interviewing in health care settings: opportunities and limitations Am J Prev Med 2001 20 1 68 74 10.1016/S0749-3797(00)00254-3 11137778

[B13] Carey K Scott-Sheldon L Carey M DeMartini K Individual-level interventions to reduce college student drinking: a meta-analytic review Addict Behav 2007 32 2469 2494 10.1016/j.addbeh.2007.05.004 17590277 PMC2144910

[B14] Larimer M Cronce J Identification, prevention, and treatment revisited: individual-focused college drinking prevention strategies 1999–2006 Addict Behav 2007 32 2439 2468 10.1016/j.addbeh.2007.05.006 17604915

[B15] Cohen J Statistical Power of Analysis for the Behavioural Sciences 1969 NY: Academic

[B16] McGue M Iacono WG Legrand LN Malone S Elkins I Origins and consequences of age at first drink. Associations with substance-use disorders, dishinbitory behavior and psychopathology, and P3 amplitude Alcohol Clin Exp Res 2001 25 8 1156 1165 10.1111/j.1530-0277.2001.tb02330.x 11505047

[B17] Bjork JM Smith AR Chen G Hommer DW Adolescents, adults and rewards: comparing motivational neurocircuitry recruitment using fMRI PLoS One 2010 5 7 e11440 10.1371/journal.pone.0011440 20625430 PMC2897849

[B18] Duke AA Giancola PR Morris DH Holt JCD Gunn RL Alcohol dose and aggression: another reason why drinking more is a bad idea J Stud Alcohol Drugs 2011 72 34 43 21138709 10.15288/jsad.2011.72.34 PMC3001679

[B19] Walton MA Chermack ST Shope JT Bingham CR Zimmerman MA Blow FC Cunningham RM Effects of a brief intervention for reducing violence and alcohol misuse among adolescents: a randomized controlled trial JAMA 2010 304 5 527 535 10.1001/jama.2010.1066 20682932 PMC3560393

[B20] Vellerman R Templeton LJ Copello A The role of the family in preventing and intervening with substance use and misuse: a comprehensive review of family interventions, with a focus on young people Drug Alcohol Rev 2005 24 93 109 10.1080/09595230500167478 16076580

[B21] Elliott G Morleo M Harkins C Cook PA Phillips-Howard PA Parents’ Perceptions of Their Children's Alcohol Consumption 2011 Liverpool John Moores University: Centre for Public Health

[B22] Medical Research Council Developing and Evaluating Complex Interventions London 2008

[B23] Lancaster G Dodd S Williamson P Design and analysis of pilot studies: recommendations for good practice J Eval Clin Pract 2004 10 2 307 312 10.1111/j..2002.384.doc.x 15189396

[B24] Thabane L Ma J Chu R Cheng J Ismaila A Rios L Robson R Thabane M Giangregorio L Goldsmith C A tutorial on pilot studies: the what, why and how BMC Med Res Methodol 2010 10 1 10 10.1186/1471-2288-10-1 20053272 PMC2824145

[B25] Kaner E Bland M Cassidy P Coulton S Deluca P Drummond C Gilvarry E Godfrey C Heather N Myles J Newbury-Birch D Oyefeso A Parrott S Perryman K Phillips T Shenker D Shepherd J Screening and brief interventions for hazardous and harmful alcohol use in primary care: a cluster randomised controlled trial protocol BMC Publ Health 2009 9 287 10.1186/1471-2458-9-287 PMC2734851 19664255

[B26] Donaldson L Guidance on the Consumption of Alcohol by Children and Young People 2009 London: Department of Health

[B27] Miller W Heather N Hall W Calculating standard drink units: international comparisons Br J Addict 1991 86 43 47 10.1111/j.1360-0443.1991.tb02627.x 2009396

[B28] NHS Health Scotland, University of Warwick, University of Edinburgh Warwick-Edinburgh Mental Well-Being Scale (WEMWBS) Scotland 2006

[B29] Knight J Sherritt L Harris S Gates E Chang G Validity of brief alcohol screening tests among adolescents: a comparison of the AUDIT, POSIT, CAGE and CRAFFT Alcohol Clin Exp Res 2003 27 1 67 73 10.1111/j.1530-0277.2003.tb02723.x 12544008

[B30] White H Labouvie H Towards the assessment of adolescent problem drinking J Stud Alcohol 1989 50 30 7 10.15288/jsa.1989.50.30 2927120

[B31] Willie N Badia X Bonsel G Burstrom K Cavrini G Devlin N Egmar A Greiner W Gusi N Herdman M Development of the EQ-5D-Y: a child-friendly version of the EQ-5D Qual Life Res 2010 19 6 875 886 10.1007/s11136-010-9648-y 20405245 PMC2892611

[B32] UKATT Research Team Cost effectiveness of treatment for alcohol problems: findings of the randomised UK alcohol treatment trial (UKATT) Br Med J 2005 331 7516 544 16150765 10.1136/bmj.331.7516.544 PMC1200587

[B33] Rollnick S Mason P Butler C Health Behaviour Change: A Guide for Practitioners 1999 Edinburgh: Churchill Livingstone

[B34] Miller W Sanchez V Motivating Young Adults for Treatment and Lifestyle Change 1993 Notre Dame, IN: University of Notre Dame Press

[B35] Department for Children, Schools and Families Working Together to Safeguard Children: A Guide to Inter-Agency Working to Safeguard and Promote the Welfare of Children London 2010

[B36] Kaner EFS Lock CA McAvoy BR Heather N Gilvarry E A RCT of three training and support strategies to encourage implementation of screening and brief alcohol intervention by general practitioners Br J Gen Pract 1999 49 699 703 10756610 PMC1313496

[B37] Coulton S Perryman K Bland M Cassidy P Crawford M Deluca P Drummond C Gilvarry E Godfrey C Heather N Kaner E Myles J Newbury-Birch D Oyefeso A Parrott S Phillips T Shenker D Shepherd J Screening and brief interventions for hazardous alcohol use in accident and emergency departments: a randomised controlled trial protocol BMC Health Serv Res 2009 9 114 10.1186/1472-6963-9-114 19575791 PMC2712466

[B38] Newbury-Birch D Bland M Cassidy P Coulton S Deluca P Drummond C Gilvarry E Godfrey C Heather N Kaner E Myles J Oyefeso A Parrott S Perryman K Phillips T Shenker D Shepherd J Screening and brief interventions for hazardous and harmful alcohol use in probation services: a cluster randomised controlled trial protocol BMC Publ Health 2009 9 418 10.1186/1471-2458-9-418 PMC2784463 19922618

[B39] Lane C Huws-Thomas M Rollnick S Hood K Edwards K Robling M Measuring adaptations of motivational interviewing: the development and validation of the Behaviour Change Counselling Index (BECCI) Patient Educ Couns 2005 56 166 173 10.1016/j.pec.2004.01.003 15653245

